# 

**Published:** 1894-05

**Authors:** 


May to July Inclusive Free.

Whole Number, CCCXCII.

New Series.

May, 1894.

VOL. XXXIII. No. 10.

LIBRARY
Indexed
S. G. O.

### Buffalo Medical and Surgical Journal.

Established 1845 by AUSTIN FLINT, M. D.

**Editors:**

THOS. LOTHROP, M. D.    WM. WARREN POTTER, M. D.

**Associate Editors:**

WILLIAM C. KRAUSS, M. D.    JAMES WRIGHT PUTNAM, M. D.
JOHN A. MILLER, Ph. D.    JOHN PARMENTER, M. D.

PUBLISHED MONTHLY.

European Agency,
TRÜBNER & CO.
57 and 59 Ludgate Hill, London, E. C.

BUFFALO:
1894.

Foreign Subscription, $2.50

Entered at the Post-Office at Buffalo, N. Y., as Second-class Mail Matter.

*Times Printing House, Buffalo, N. Y.*

SUBSCRIPTION PRICE, $2.00 IF PAID IN ADVANCE; OTHERWISE $2.50 A YEAR.

Table of Contents on Page V.

